# Mentalising music in frontotemporal dementia

**DOI:** 10.1016/j.cortex.2012.09.011

**Published:** 2013-07

**Authors:** Laura E. Downey, Alice Blezat, Jennifer Nicholas, Rohani Omar, Hannah L. Golden, Colin J. Mahoney, Sebastian J. Crutch, Jason D. Warren

**Affiliations:** Dementia Research Centre, UCL Institute of Neurology, University College London, London, United Kingdom

**Keywords:** Mentalising, Theory of mind, Music, Frontotemporal dementia

## Abstract

Despite considerable recent interest, the biological basis and clinical diagnosis of behavioural variant frontotemporal dementia (bvFTD) pose unresolved problems. Mentalising (the cognitive capacity to interpret the behaviour of oneself and others in terms of mental states) is impaired as a prominent feature of bvFTD, consistent with involvement of brain regions including ventro-medial prefrontal cortex (PFC), orbitofrontal cortex and anterior temporal lobes. Here, we investigated mentalising ability in a cohort of patients with bvFTD using a novel modality: music. We constructed a novel neuropsychological battery requiring attribution of affective mental or non-mental associations to musical stimuli. Mentalising performance of patients with bvFTD (*n* = 20) was assessed in relation to matched healthy control subjects (*n* = 20); patients also had a comprehensive assessment of behaviour and general neuropsychological functions. Neuroanatomical correlates of performance on the experimental tasks were investigated using voxel-based morphometry of patients' brain magnetic resonance imaging (MRI) scans. Compared to healthy control subjects, patients showed impaired ability to attribute mental states but not non-mental characteristics to music, and this deficit correlated with performance on a standard test of social inference and with carer ratings of patients' empathic capacity, but not with other potentially relevant measures of general neuropsychological function. Mentalising performance in the bvFTD group was associated with grey matter changes in anterior temporal lobe and ventro-medial PFC. These findings suggest that music can represent surrogate mental states and the ability to construct such mental representations is impaired in bvFTD, with potential implications for our understanding of the biology of bvFTD and human social cognition more broadly.

## 1 Introduction

Frontotemporal lobar degeneration (FTLD) refers to a group of diseases collectively characterised by atrophy of the frontal and temporal lobes. The most common syndrome of FTLD, behavioural variant frontotemporal dementia (bvFTD), manifests as progressive behavioural decline leading to severe social dysfunction, as reflected in recent consensus diagnostic criteria (Rascovsky et al., 2011). The bvFTD syndrome presents important neurobiological and clinical problems. The neurobiological basis for the selective erosion of neural circuitry mediating complex behaviours in bvFTD remains poorly understood, while early diagnosis is often difficult due to the insidious nature of behavioural decline, potential confusion with primary psychiatric illness and phenotypic overlap with other dementia diseases (Gregory et al., 2002; Snowden et al., 2003; Balsis et al., 2005). Accordingly, there is considerable interest in identifying novel metrics of bvFTD that might illuminate underlying mechanisms and potentially facilitate diagnosis. An important emerging theme in the neurobiology of bvFTD is the concept of a selectively vulnerable, large-scale brain network including prefrontal cortex (PFC), orbitofrontal cortex (OFC), anterior cingulate, insula and their projections: this network is likely to be fundamentally concerned with social cognitive processing and the signature of network involvement may separate bvFTD from other neurodegenerative disorders (Seeley et al., 2007, 2012; Zhou et al., 2010, 2012; Raj et al., 2012). This evidence suggests that biomarkers that can capture network characteristics might be diagnostically useful, and that network function in bvFTD might be best assessed using indices of complex social behaviours.

Mentalising can be broadly defined as the cognitive capacity by which we interpret the behaviour of oneself and others in terms of mental states (Frith and Frith, 2003). The term ‘theory of mind’ (ToM) is often used interchangeably with mentalising, but can be defined more precisely as a crucial component of the mentalising process whereby mental states are explicitly attributed to others (Robbins, 2004). ToM and mentalising in the broader sense together constitute a key capacity within the wider domain of social cognition. These complex cognitive functions require the representation, analysis and integration of a variety of social signals. ToM capacity can be further subclassified as ToM for the attribution of beliefs and intentions (‘cognitive’ ToM) and ToM for the attribution of feeling states (‘affective’ ToM), though these separable capacities frequently interact in everyday life (Poletti et al., 2012). Widely used tests of ToM such as the ‘Mind in the Eyes’ task (Baron-Cohen et al., 2001) largely index affective ToM using stimuli derived from other humans, however it has been repeatedly shown that intentionality can be attributed even to abstract, inanimate stimuli (e.g., cartoon shapes: Heider and Simmel, 1944; Berry and Springer, 1993; Castelli et al., 2000; Blakemore et al., 2003). Neuroimaging studies in healthy individuals have linked the ability to mentalise with a network of brain regions, in particular ventro-medial PFC and frontal pole, OFC (Gallagher and Frith, 2003; Carrington and Bailey, 2009; Moll et al., 2011; Abu-Akel and Shamay-Tsoory, 2011) and the anterior temporal lobes (Fumagalli and Priori, 2012). The study of disease states potentially allows identification of brain areas critical for ToM. Impaired ToM occurs on a developmental basis as a hallmark of autism (Baron-Cohen et al., 1985, 1999) and may also develop in association with a variety of focal brain lesions (Martin-Rodriguez and Leon-Carrion, 2010). Deficits of ToM in neurodegenerative disease have attracted much recent attention and on clinical and neuroanatomical grounds may be particularly relevant to bvFTD (Schroeter, 2012; Poletti et al., 2012). Patients with bvFTD frequently have difficulty with aspects of social cognition that are likely to be relevant to ToM, including emotion recognition (Rosen et al., 2005; Kipps et al., 2009b; Omar et al., 2011), empathic concern and perspective taking (Lough et al., 2006; Rankin et al., 2006; Eslinger et al., 2011), and perception of humour and sarcasm (Snowden et al., 2003; Kosmidis et al., 2008; Kipps et al., 2009b). A specific mentalising deficit may be an early feature of bvFTD (Gregory et al., 2002; Adenzato et al., 2010) and neuroanatomical substrates for this deficit have been proposed. The distributed neural network that is damaged in bvFTD (Seeley et al., 2007; Zhou et al., 2010, 2012; Raj et al., 2012) overlaps brain areas previously implicated in ToM (Gallagher and Frith, 2003; Carrington and Bailey, 2009). Impaired ability to experience social emotions has been linked to frontopolar damage in bvFTD (Moll et al., 2011). In addition, bvFTD is often associated with damage involving anterior temporal lobe regions that represent social concepts underpinning normal mentalising (Zahn et al., 2009): these anterior temporal areas interact with medial PFC during moral reasoning (Fumagalli and Priori, 2012), while anterior temporal lobe damage has been implicated in the pathogenesis of cognitive and affective ToM deficits in another FTLD syndrome, semantic dementia (Duval et al., 2012).

Relations between mentalising, ToM and music processing have not been widely studied; however, music is likely a priori to engage brain processes relevant to ToM and it is an attractive candidate stimulus for probing such processes in bvFTD. Music typically entails decoding of an emotional ‘message’ and music-making generally has a strong social context across human societies (Mithen, 2005; Levitin, 2007). Music has been shown to modulate semantic information in other cognitive systems, such as language (Koelsch et al., 2004). Deficits in processing emotion information in music have been demonstrated in various disease states, notably the frontotemporal dementias, and are dissociable from the processing of other kinds of musical perceptual information (Stewart et al., 2006; Omar et al., 2010, 2011; Johnson et al., 2011; Hsieh et al., 2012). The brain mechanisms of music processing in health and disease and the brain substrates for processing emotional information in music have received considerable attention (Blood et al., 1999; Blood and Zatorre, 2001; Griffiths et al., 2004; Gosselin et al., 2006; Koelsch et al., 2006; Mitterschiffthaler et al., 2007; Mizuno and Sugishita, 2007; Caria et al., 2011; Brattico et al., 2011). Previous work has implicated a distributed network of cortical and subcortical (in particular, limbic) areas in mediating the emotional response to music, suggesting that music processing unites cognitive representational and evaluative mechanisms with the more ‘primitive’ neural mechanisms of reward and biological drives (Blood and Zatorre, 2001; Salimpoor et al., 2011; Omar et al., 2011). From this perspective, music might therefore be regarded as a comprehensive and biologically relevant model stimulus for assessing human frontal lobe functions.

More specifically, recognition of emotion in music engages prefrontal and anterior temporal components of the brain network previously implicated in ToM processing (Blood et al., 1999; Rankin et al., 2006; Mizuno and Sugishita, 2007; Zahn et al., 2007, 2009; Brattico et al., 2011; Eslinger et al., 2011) and damage involving this network has been linked specifically to deficits of music emotion recognition as well as ToM in bvFTD (Omar et al., 2011; Hsieh et al., 2012; Poletti et al., 2012). Most previous studies of music emotion processing in the normal brain and in disease states have assessed the processing of elementary or canonical emotions (e.g., ‘happiness’, ‘sadness’, ‘anger’) or basic affective dimensions such as consonance – dissonance in music (e.g., Gosselin et al., 2006; Koelsch et al., 2006; Mitterschiffthaler et al., 2007; Omar et al., 2010, 2011; Caria et al., 2011; Brattico et al., 2011). There is a sense in which all emotional attributions to music involve some degree of mentalising, since musical emotions must be inferred rather than existing explicitly in the stimuli as do animate emotions in facial and vocal expressions. However, behavioural and neuroimaging findings in autism and other disorders of social conduct suggest that music has complex interactions with mentalising (Bhatara et al., 2009; Heaton and Allen, 2009; Caria et al., 2011). In particular, it has been demonstrated directly that normal listeners are able to make mentalising judgements about composer agency from musical pieces, and such judgements have functional magnetic resonance imaging (MRI) correlates in the same medial prefrontal and anterior temporal network mediating other kinds of ToM attributions (Steinbeis and Koelsch, 2009). Music is an abstract stimulus yet is widely accessible and highly effective in conveying certain kinds of emotional signals: whereas actual social interactions are often highly complex with many potentially relevant variables, music might allow such interactions to be presented in a reduced, surrogate form that isolates elements critical for mentalising (Warren, 2008). In particular, music may code multi-component or ambiguous feeling states as abstract representations. Taken together the available evidence suggests that music may probe the interface between ToM, knowledge of social concepts and biological reward (Blood and Zatorre, 2001; Omar et al., 2011); this complex interface is characteristic of ‘real world’ social interactions, but difficult to access using conventional neuropsychological stimuli.

In this study we assessed mentalising in music using a novel paradigm based on the attribution of affective mental states in a cohort of patients with bvFTD and in healthy older control subjects. Neuroanatomical correlates of mentalising ability in the patient group were assessed using voxel-based morphometry (VBM) on structural brain MRI data. Based on previous evidence concerning ToM processing in FTLD (Gregory et al., 2002; Kipps and Hodges, 2006; Adenzato et al., 2010), we hypothesised that attribution of mental states (but not other kinds of attributions) to musical stimuli would be selectively vulnerable in bvFTD. We further hypothesised that performance on the mentalising task would correlate with grey matter volume in medial PFC, OFC and anterior temporal regions previously implicated in both ToM and emotion recognition in music, in FTLD and in the healthy brain (Menon and Levitin, 2005; Zahn et al., 2007, 2009; Steinbeis and Koelsch, 2009; Eslinger et al., 2011; Omar et al., 2011).

## 2 Methods

### 2.1 Subjects

Twenty consecutive patients fulfilling consensus criteria for bvFTD (Rascovsky et al., 2011) were recruited from the tertiary-level Specialist Cognitive Disorders Clinic at the National Hospital for Neurology and Neurosurgery, London, United Kingdom (details summarised in Table 1). All bvFTD patients had structural MRI evidence of frontal lobe atrophy with or without accompanying temporal lobe atrophy, in support of the syndromic diagnosis of bvFTD. Twenty healthy control subjects with no history of neurological or psychiatric illness were also recruited (Table 1). No subject had a history of clinically significant hearing loss. All subjects had an assessment of general neuropsychological functions (Table 1), including the Awareness of Social Inference Test (TASIT; McDonald et al., 2003). Patients' carers completed the Cambridge Behavioural Inventory (CBI; Wedderburn et al., 2008) as an index of behavioural symptoms; item 78 on the CBI (‘Appears indifferent to the worries and concerns of family members’) was selected for further analysis as the item most relevant to ToM. All participants were native to Britain, except one subject who had been resident within the United Kingdom for 15 years, and all had lifelong exposure to Western music. Most subjects had fewer than two years formal music training, corresponding to the ‘least trained’ (novice, non-musician) category of musical experience described by Halpern et al. (1995). Informed consent was obtained for all subjects and the study was approved by the local research ethics committee under Declaration of Helsinki guidelines.

### 2.2 Experimental behavioural assessment

#### 2.2.1 Structure of the experimental test

In order to assess mentalising on musical stimuli, we designed a novel behavioural paradigm to assess affective ToM based on forced-choice cross-modal matching of musical samples to word–picture combinations. The paradigm incorporated two conditions which were administered sequentially as separate subtests. In the ‘mentalising’ condition, music stimuli represented particular affective mental states. In the other ‘non-mentalising’ condition (designed as a control for the ‘mentalising’ condition), music stimuli represented non-mental objects and events. Music stimuli were all short non-vocal excerpts derived from the Western classical corpus, including solo instrumental, chamber and orchestral pieces; the complete list of stimuli and foils for each subtest is presented in Supplementary material on-line (Table S1). Musical excerpts were selected from the longer source piece based on the effectiveness of the particular excerpt in representing the mental state or the non-mental object or event, rather than from a fixed section or segment of every source piece (examples of the stimuli are available from the authors). On each trial, the task was to decide which of three word–picture combinations best described the musical sample; each word-picture triad comprised the target, a close foil and a more distant foil (for example, in the mentalising condition, ‘dreamy’ [target] – ‘dreading’ [close foil] – ‘adventurous’ [distant foil]; in the non-mentalising condition, ‘raindrops’ [target] – ‘birdcall’ [close foil] – ‘train’ [distant foil]). In the mentalising condition, stimuli and foils were designed to reduce reliance on elementary emotion judgements that could be based on simple perceptual cues (for example, ‘dreamy’ does not have a close elementary emotional analogue, and would not be distinguished from the close foil ‘dreading’ based on a single perceptual cue such as ‘slow tempo’); the word choices were in most cases synonyms of those used to designate affective mental states in a standard test of ToM, the Baron-Cohen ‘Reading the Mind in the Eyes’ test (Baron-Cohen et al., 2001).

Music stimuli for both conditions were chosen based on pilot data in a separate group of 25 young healthy control subjects; all musical samples included in the final test were matched to the target word–picture combination by at least 80% of subjects in the pilot control group (further details of the pilot study are provided in Supplementary Material on-line). As a further criterion used in selecting musical examples for the pilot study, we avoided pieces with strong prior semantic associations (in particular, descriptive titles) likely to be widely familiar to musically untrained listeners and implying by association a particular mental or non-mental representation. The musical stimulus sets in the mentalising and non-mentalising conditions were closely comparable in duration, tempo, harmonic and timbral characteristics (solo instrument, chamber or orchestral texture – see Table S1). Pictorial stimuli for the matching task were selected from public Internet databases.

#### 2.2.2 Experimental procedure

The experimental test was administered under Matlab7^©^ (www.mathworks.com) running the Cogent1.25 toolbox (www.vislab.ucl.ac.uk/cogent) on a notebook computer. Music stimuli were presented in free-field at a comfortable listening level for each subject (at least 70 dB). Subjects were first familiarised with the paradigm using musical examples not subsequently presented in the actual test. Twenty test trials were administered in each condition; conditions were presented in fixed order (non-mentalising followed by mentalising). Combinations of words and pictures (high quality colour images) were simultaneously presented on the computer monitor. Trials were presented in a fixed randomised order, and the relative screen positions of targets and foils were randomised from trial to trial. Subject selections were recorded and stored for offline analysis. In addition, on each trial the subject was asked if they were familiar with the piece, and this information was also recorded. Each piece was presented once; a single repeat of a trial was allowed if the examiner considered that the subject had been distracted during the original presentation. No time limit was imposed and no feedback about performance was given during the test.

#### 2.2.3 Analysis of behavioural data

Behavioural data were analysed using STATA 12^©^. Experimental data were analysed using analysis of variance (ANOVA) regression models incorporating subject scores in the mentalising or non-mentalising condition as a within-subject variable, group (bvFTD or control) as a between-subjects variable; and subject age, gender, and scores on the colour-word inhibition Stroop task, the British Picture Vocabulary Scale (BPVS; Lloyd et al., 1982), and the National Adult Reading Test (NART) as covariates of no interest (to adjust for possible performance effects of demographic bias, general executive capacity, single-word comprehension, and premorbid IQ, respectively). Imageability and lexical frequency of the words presented in both conditions were calculated using the N-Watch psycholinguistic research database (http://www.pc.rhul.ac.uk/staff/c.davis/Utilities/), in order to examine whether such characteristics could be contributing to the results. Population averaged models for repeated measures were used to examine the group by task interaction, with and without adjustment for word imageability and lexical frequency.

In order to assess how well mentalising and non-mentalising conditions were able to discriminate bvFTD patients from healthy controls we constructed receiver operating characteristic (ROC) curves whereby the discriminatory ability of each task was quantified using the area under the curve (AUC). The AUC is the probability that in a randomly selected patient/control pair, the patient has a lower score than the control (Hanley and McNeil, 1982); perfect discrimination between patient and control groups would correspond to an AUC of 1, whilst the same distribution of scores in patients and controls would correspond to an AUC of .5. Correlations between experimental tasks and between each experimental task and potentially relevant demographic, behavioural (CBI) and general neuropsychological variables were assessed using Spearman's rho in the patient group.

### 2.3 Brain image acquisition and analysis

#### 2.3.1 Image acquisition and pre-processing

At the time of behavioural assessment each patient had volumetric brain MRI on a 3.0 T GE Signa scanner (General Electric, Milwaukee, Wisconsin, USA) using a standard quadrature head coil. *T*_1_-weighted volumetric images were obtained with a 24 cm field of view and 256 × 256 matrix to provide 124 contiguous 1.5 mm thick slices in the coronal plane (echo time (TE) = 5 msec, repetition time (TR) = 512 msec, inversion time (TI) = 5650 msec). Three patients were unable to tolerate a scan, and one scan was heavily degraded by movement artefact, resulting in a total of 16 scans from the bvFTD cohort suitable for entry into the VBM analysis.

Pre-processing of patient brain MR images was performed using the DARTEL toolbox of SPM8 (www.fil.ion.ucl.ac.uk/spm) running under Matlab 7.0 (Ridgway et al., 2008). Normalisation, segmentation, modulation and smoothing of grey and white matter images were performed using default parameter settings. In order to adjust for individual differences in global grey matter volume during subsequent analysis, total intracranial volume (TIV) was calculated for each participant by summing grey matter, white matter and cerebrospinal fluid volumes following segmentation of all three tissue classes. A study-specific template brain image was created by warping all native space whole-brain images to the final DARTEL template and calculating the average of the warped brain images.

#### 2.3.2 Image analysis

Linear regression models were used to examine regional grey matter volume correlated with performance on each of the experimental subtests; voxel intensity (grey matter volume) was modelled as a function of subtest score across the group, including participant's age, TIV and Stroop inhibition score (a measure of general executive performance) as covariates of no interest. Separate models were used to assess grey matter associations of each experimental task separately and after combining task regressors in a common design matrix (to allow neuroanatomical associations of each task to be compared directly). To help protect against voxel drop-out because of potentially marked local regional atrophy in particular scans, we applied a customised explicit brain mask based on a specified ‘consensus’ voxel threshold intensity criterion (Ridgway et al., 2009) whereby a voxel was included in the analysis if grey matter intensity at that voxel was >.1 in >70% of the participants [rather than in all participants, as with the default SPM8 mask. Statistical parametric maps (SPMs) of regional grey matter volume correlating with score on each experimental subtest were examined at threshold *p* < .05 after family-wise error (FWE) correction for multiple comparisons over the whole brain and after small volume correction using anatomical regions based on our a priori hypotheses.

Anatomical small volumes were derived by manual tracing from the template brain image using MRIcron^®^ (http://www.sph.sc.edu/comd/rorder/mricron.html) and comprised a prefrontal (combined OFC – ventro-medial PFC) region and left and right anterior temporal lobe regions. The prefrontal region included bilateral OFC (including the orbital surface of both frontal lobes and the lateral orbital gyri below the inferior frontal sulcus bilaterally) and ventro-medial PFC (the medial inter-hemispheric surface of both frontal lobes, extending superiorly to the apex of the callosal genu). Each anterior temporal lobe volume extended from the temporal pole posteriorly to the most anterior extension of Heschl's sulcus (Kim et al., 2000). These volume boundaries were intentionally generous, to ensure that individual variations in brain anatomy were all fully encompassed, however, all anatomical attributions within these volumes were subsequently checked visually in order to ensure accurate localisation to particular regions within the volume.

SPMs were displayed as overlays on the study-specific template brain image. An additional cluster extent threshold of 50 voxels was applied when reporting significant clusters.

## 3 Results

### 3.1 Demographic characteristics

The bvFTD and control groups were well matched for age (*t*_38_ = .42, *p* = .7); males were over-represented in the patient group (*t*_40_ = 2.7, *p* = .009), and gender accordingly was included as a covariate of no interest in all analyses. Patients and controls did not differ significantly in educational background, though there was a trend to longer time spent in formal education in the control group (*t*_38_ = 1.94, *p* = .06).

### 3.2 General neuropsychological performance

As a group the bvFTD patients showed the anticipated profile of deficits relative to healthy control subjects, with impaired performance on measures of executive function, memory and naming (Table 1). Relative to this control group (who displayed superior neuropsychological performance), patients also showed reduced single-word comprehension and visual object perception. However, it is of note that these scores did not fall within the impaired range (<5th percentile) based on published norms.

### 3.3 Experimental task performance

**Fig. 1:**
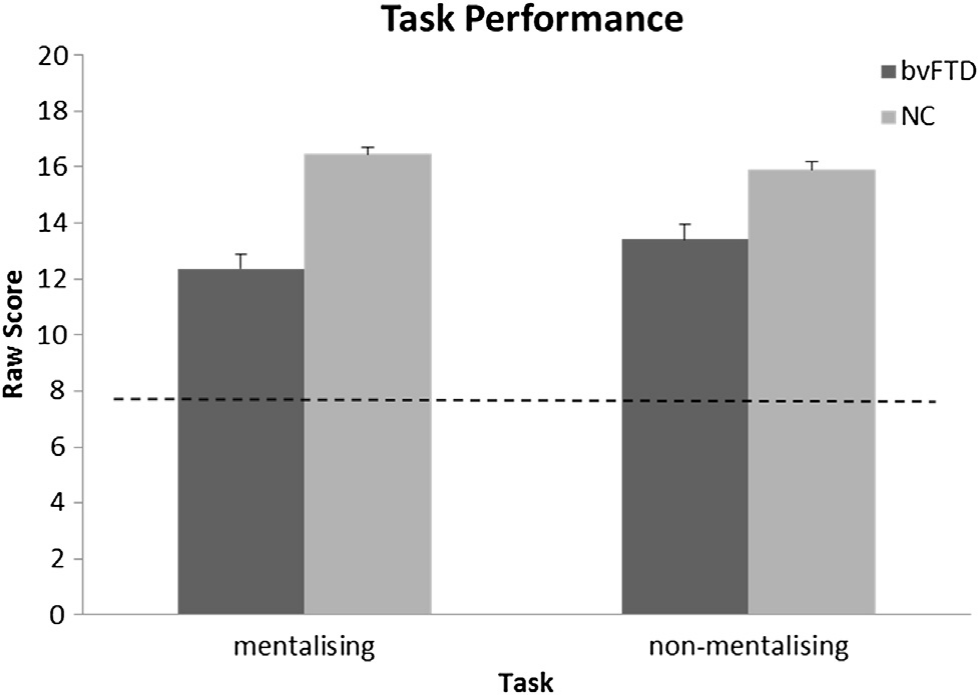
Performance [mean + standard error of mean (S.E.M.)] on musical mentalising and non-mentalising tasks for the bvFTD and normal control (NC) groups. Broken line represents chance score.

Scores for the bvFTD and control groups in each subtest are displayed in Fig. 1. Healthy control subjects performed comparably on both experimental tasks. A repeated measures ANOVA regression model (adjusted for verbal comprehension, general executive performance, premorbid IQ, age, and gender) revealed a significant deficit in the bvFTD group relative to healthy controls in the mentalising condition (*F*_6,29_ = 6.45, *p* < .003); there was no significant group performance difference in the non-mentalising condition, though there was a non-significant trend to worse performance in the bvFTD group on this subtest (*F*_6,29_ = 2.45, *p* = .08). No significant group by task interaction was found. However, after adjustment for word imageability and frequency the estimated group by task interaction was of similar magnitude, suggesting that these components had little impact on the findings.

Spearman correlation analyses revealed that scores on the two experimental tasks were significantly correlated (rho = .54, *p* = .003). Scores on the TASIT were found to be significantly selectively correlated with performance on the mentalising task, (rho = .55, *p* = .002) though not the non-mentalising task (rho = .34, *p* = .067). In addition, scores on the selected CBI item (‘Appears indifferent to the worries and concerns of family members’) were significantly negatively correlated with performance on the mentalising task (rho = −.6, *p* = .03), but not the non-mentalising task (rho = −.1, *p* = .67). There were no correlations of performance on either experimental task with executive function, single-word comprehension, clinical disease duration, years of education, or premorbid intelligence estimates. Only two control subjects reported prior familiarity with over half the musical examples used; most participants reported no prior familiarity with the musical examples. Accordingly we did not perform a formal regression analysis of performance on prior musical familiarity. However, a separate analysis excluding the two control subjects who reported higher prior familiarity with the musical examples yielded identical results with respect to the experimental tasks.

**Fig. 2:**
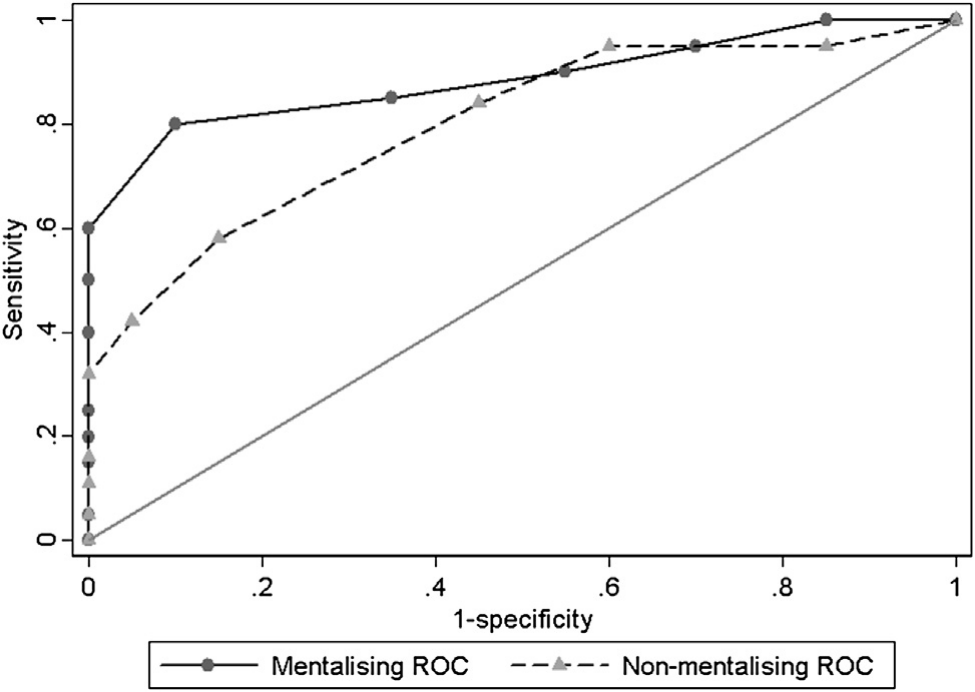
Comparison of musical mentalising and non-mentalising performance AUC: prediction of disease by task performance. The ROC curves use total scores (/20) in each task to discriminate between bvFTD patients and NCs.

ROC curves based on each of the experimental tasks discriminated between bvFTD patients and healthy controls (Fig. 2). No significant AUC difference was found between the mentalising and non-mentalising tasks, however mentalising task performance showed a trend towards greater sensitivity and specificity (AUC coefficient .88 [95% confidence interval (CI): .73, .95]) compared with the non-mentalising task (AUC coefficient .73 [95% CI: .57, .90]). Further binomial breakdown of the AUCs revealed that a cut-point raw score of 15 on the mentalising task correctly classified 85% of participants as being either a patient or a control, whereas this was reduced to 71% for the non-mentalising task using the same cut-point value.

**Fig. 3:**
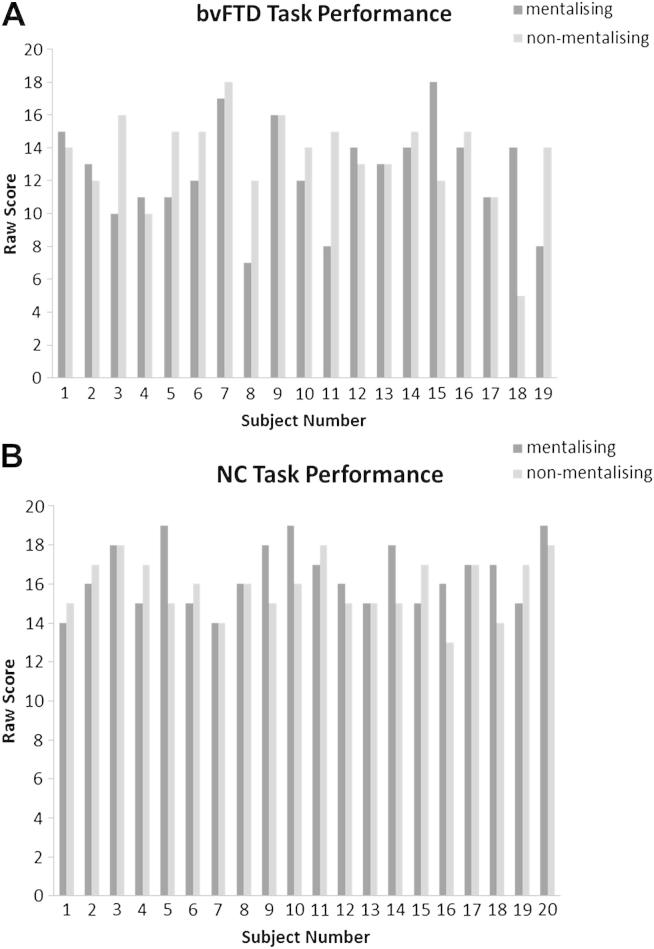
Individual performance profiles for subjects in the bvFTD and NC groups. (A). Patients 3, 5, 8, 11 and 19 showed a >four point discrepancy between subtest scores, with superior performance on the musical non-mentalising task; patients 15 and 18 showed the reverse performance pattern. (B) For individuals in the NC group, the discrepancy between scores on each subtest was never >three points.

Examining individual subject performance profiles (Fig. 3), five patients showed a clear (>four point) discrepancy in favour of superior performance on the non-mentalising task. However, two patients showed the reverse pattern, with superior performance on the mentalising task. No similarly marked discrepancies were seen for individuals in the healthy control group (Fig. 3).

### 3.4 Neuroanatomical associations

**Fig. 4:**
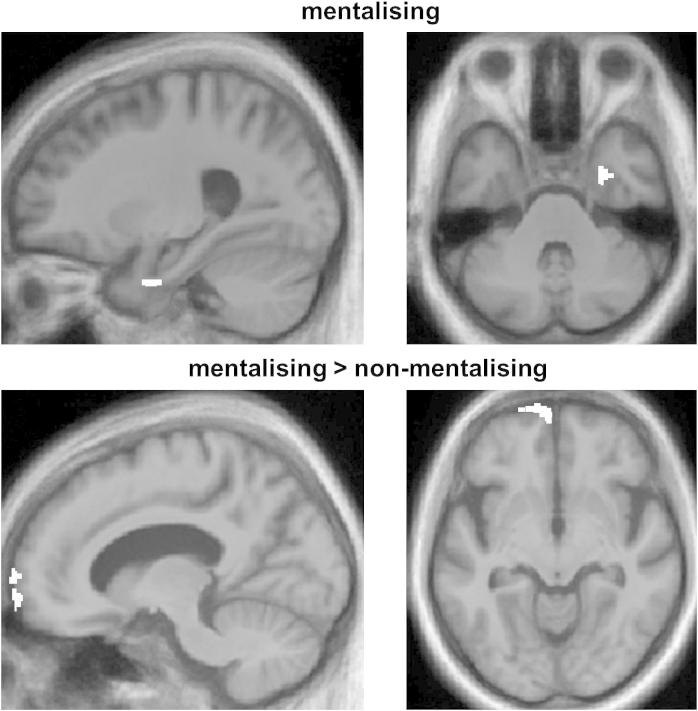
SPMs (shown in white) of regional grey matter volume positively correlated with performance on the musical mentalising task (above) and significantly more strongly correlated with performance on the mentalising than the non-mentalising task (below) in the bvFTD group. SPMs are rendered on the mean customised template image in Montreal Neurological Institute standard stereotactic space; sagittal (left) and axial (right) sections are shown, and the left hemisphere is shown on the left in axial sections. For display purposes, SPMs have been thresholded at *p* < .001 uncorrected; however, the grey matter correlations shown were also present thresholded at *p* < .05 after correction for multiple comparisons (see Table 2).

SPMs of grey matter volume associated with performance in the mentalising and non-mentalising conditions are shown in Fig. 4; data for local maxima of grey matter change are summarised in Table 2. When assessed separately, performance on the mentalising task was positively associated with grey matter volume in right entorhinal cortex (*p* < .05 after FWE correction for multiple comparisons within the anatomical small volume of interest). No significant negative inverse associations between performance and grey matter volume were identified. Performance on the non-mentalising task showed no significant positive associations with grey matter volume at the prescribed threshold. However, performance on the non-mentalising task was inversely associated with grey matter volume in ventro-medial PFC (*p* < .05 after FWE correction for multiple comparisons over the whole-brain). When neuroanatomical associations of performance in each task were compared in a combined design, performance on the mentalising task was significantly more strongly associated with grey matter in ventro-medial PFC than was performance on the non-mentalising task (*p* < .05 after FWE correction for multiple comparisons over the whole-brain); no significant grey matter associations of the reverse contrast were identified.

## 4 Discussion

Here we have presented evidence that ability to attribute surrogate affective mental states to music is impaired in bvFTD. These findings move beyond previous work demonstrating that the ability to label simple emotions in music is impaired in bvFTD as part of a more general multimodal impairment of emotion processing (Omar et al., 2011; Hsieh et al., 2012): the deficit demonstrated here lay with attribution of more complex feeling states to music, and furthermore, the deficit was at least partly specific for the attribution of mental states versus other, non-mental representations within the domain of music. This musical mentalising deficit was not attributable to general executive dysfunction, lower premorbid intelligence or other potentially relevant confounding factors, but did correlate specifically with performance on a test of social inference (TASIT) requiring interpretation of others' mental states, as well as with carer-reported real-world quantitative estimates of patients' ability to interpret others' mental states on the CBI (an index shown previously to be sensitive to functional behavioural changes in bvFTD: [Kipps et al., 2009a]). We cannot completely exclude the possibility that performance on the musical mentalising task was driven by processing of word and picture labels rather than musical pieces per se: however, a selective musical mentalising deficit was demonstrated after adjusting for certain relevant characteristics of the labels in each condition and adjusting for general verbal semantic capacity. The specific correlation of experimental mentalising task performance here with standard measures of mentalising performance provides further evidence that our mentalising task here did, indeed, index musical mentalising capacity. The relative specificity of the mentalising deficit shown by our patients is in keeping with previous evidence that patients with bvFTD can exhibit dissociable impairments of ToM function independent of general executive capacity (Lough et al., 2001). The present findings show that, remarkably, the mentalising deficit in bvFTD extends to the abstract realm of music. Because music is a somewhat unusual vehicle for attributions of this kind, the question arises whether the results could simply reflect a task difficulty effect. This explanation is unlikely: attribution of non-mental characteristics to music is, if anything, intrinsically even more demanding, and indeed, single subject performance profiles here revealed individual patients who had selective difficulty with non-mental musical attributions. Furthermore, healthy control subjects showed no such task-specific effect.

The behavioural mentalising deficit here was associated with grey matter changes in brain regions (the anterior temporal lobe and ventro-medial PFC) previously implicated in mentalising both in the healthy brain and in disease (Gallagher and Frith, 2003; Carrington and Bailey, 2009). In particular, the anterior medial prefrontal and right anterior temporal cortical associations here were in proximity to areas identified in a previous study of mentalising in music (Steinbeis and Koelsch, 2009). Furthermore, the neuroanatomical associations we have identified are in line with previous evidence for the brain substrates of mentalising in other modalities in bvFTD (Gregory et al., 2002; Kipps et al., 2009b). The positive correlation of grey matter in anterior temporal cortex with musical mentalising ability accords with previous evidence that this region abstracts information relevant to social concept processing (Zahn et al., 2009). The inverse correlation of grey matter in PFC with performance in the non-mentalising condition may imply that relative sparing of mentalising regions (in the context of more widespread associated brain damage) interferes with analysis of music for non-mental representations. Atrophy of inferior frontal lobe cortex has previously been shown to be an early feature of bvFTD (Perry et al., 2006): though detailed longitudinal behavioural studies are presently lacking, a strong prima facie case could be made on both clinical and neuroimaging grounds that mentalising ability may be a sensitive and early indicator of incipient bvFTD. Caution is needed in interpreting the present VBM results, since the patient cohort was relatively small in relation to the known clinical and anatomical heterogeneity of bvFTD (Rohrer et al., 2011). However, acknowledging this caveat, we would argue based on the present evidence that music is a promising model system to capture ToM dysfunction and perhaps thereby assist in the early detection of bvFTD: musical mentalising requires representation of abstract qualities from a complex stimulus, for which (unlike real-life social scenarios) stimulus properties can be manipulated relatively precisely.

Aside from their clinical implications, our findings speak to certain key issues in the neurobiology of music and social cognition more generally. The neurobiological study of music is challenging, as there are currently no adequate non-human models of music processing and music is typically invested with extensive socio-cultural associations that are at least partly learned. Indeed, music constitutes a universal human artefact that (along with other artefacts) can be invested with social signals such as agency (Steinbeis and Koelsch, 2009). Functional imaging of the healthy brain can delineate correlates of music processing but cannot distinguish critical correlates from those that may be epiphenomenal. Human diseases that affect music processing therefore constitute potentially informative ‘experiments of nature’; however, most diseases produce substantial associated brain damage impacting on non-musical functions or (like stroke) they affect musical processing mechanisms stochastically. bvFTD is an ideal model system with which to address core biological functions of music: this disease selectively affects complex human social behaviours while sparing many other aspects of cognition, and targets a large-scale intrinsic brain network that links sensory experience with affective, semantic and reward processing (Seeley et al., 2007; Zhou et al., 2010, 2012; Raj et al., 2012). It has been demonstrated that neural structures predominantly implicated in bvFTD include long Von Economo projection neurons linking insular, cingulate and prefrontal cortices and subcortical centres (Seeley et al., 2012). Humans are one of a small number of species that possess these neurons and they appear to serve as a critical substrate for complex social behaviour. The network bound by these neurons has also been shown to be integral to music processing (Blood and Zatorre, 2001; Omar et al., 2011). Previously this was somewhat paradoxical, as the evolutionary value of music remains speculative (Mithen, 2005). The present findings in bvFTD raise the possibility that the modelling of mental states may be a core neurobiological function of music.

This interpretation is in line with accumulating neurobiological and ethnographic evidences (Levitin, 2007). It has been proposed that music played a specific role in decoding others' emotion states during human evolution (Mithen, 2005). Recognition of emotion in music engages components of the brain network previously implicated in mentalising (Rankin et al., 2006; Zahn et al., 2007, 2009; Eslinger et al., 2011) and behavioural findings in autism and other disorders of social conduct have previously suggested that music influences mentalising (Bhatara et al., 2009; Heaton and Allen, 2009). We propose that, precisely on account of its abstract, inanimate nature, music may be highly effective in conveying certain kinds of signals relevant to mentalising: whereas actual social interactions are often highly complex with many potentially relevant variables, music might allow such interactions to be presented in a reduced, surrogate form that isolates elements critical for mentalising with low behavioural cost (Warren, 2008). A capacity to use music in this way would likely enhance empathy and pair-bonding and might therefore have been selected during human evolution (Mithen, 2005; Warren, 2008). This hypothesis requires further examination with a more detailed behavioural examination of musical mentalising in the healthy brain as well as disease states, and with correlative structural and functional imaging to establish the underlying brain mechanisms.

This study has several limitations. There is a need to further substantiate the validity and limitations of the mentalising paradigm as applied to music, and the relation between elementary emotion processing and the attribution of more complex or ambiguous affective states to music. There is no universally agreed ‘lexicon’ of musical emotions and more information is needed about musical mentalising in the healthy brain. Mentalising in music should ideally be studied in the context of a comprehensive assessment of mentalising abilities in different modalities; this would enable evaluation of the specificity and sensitivity of the music-associated deficit. In a related vein, it would be relevant to manipulate musical stimulus parameters such as familiarity, valence and complexity as well as perceptual characteristics to assess the extent to which these may modulate mentalising on musical stimuli. From a clinical perspective, there is a need for detailed neuropathological correlation in bvFTD populations, both to establish disease associations and to correlate behavioural deficits with histopathological features. It would, for example, be intriguing to evaluate the role of Von Economo neurons in this very specifically human ability (Seeley et al., 2012). In addition, the promise of early disease detection requires further substantiation of the timing of development of mentalising deficits in the course of bvFTD evolution. It would be of great interest, for example, to establish whether such deficits (and particularly, deficits of more abstract ToM processes, such as those embodied in music) might lead other features in presymptomatic carriers of mutations causing bvFTD. This will require longitudinal study of individuals affected and at-risk of developing bvFTD. Taking these caveats into account, the present findings provide evidence that music can represent surrogate mental states and that the ability to construct such mental representations is impaired in bvFTD. The findings have potential implications for our understanding of the biology of this disease and human social cognition more broadly.

## Author contributions

LED, CJM, SJC and JDW were each involved in designing and conducting the study, and in drafting and critically revising the manuscript. LED collected and analysed the data and CJM also provided technical assistance with the neuroimaging analysis. AB and RO created the stimulus sets, collected pilot data and were involved in drafting the manuscript. HLG assisted with collection of neuropsychological data and was involved in drafting the manuscript. JN designed and supervised the statistical analysis and was involved in drafting the manuscript.

## Competing interests

LED, CJM, HLG, SJC and JDW receive salary and research support from the Medical Research Council, Alzheimer Research UK and the Wellcome Trust.

We have no financial competing interests to declare.

## Figures and Tables

**Table 1:** Demographic and neuropsychological characteristics of bvFTD patients and healthy control subjects.

	bvFTD^c^	<10th percentile^d^	Controls	*p* value
No.	20		20	n/a
Age (years)	64 (9.3)		65 (8.5)	.70
Sex (F:M)	4:16		11:9	**.009**
Education (years)	13 (3)		15 (2)	.06
Non-musicians^b^	19		19	ns
CBI (/324)	110 (55)		n/a	n/a
CBI (item 78; /4)^a^	2.25 (1.5)		n/a	n/a
Symptom duration (years)	5.5 (5.4)		n/a	n/a

**IQ**				
WASI vocab	46.16 (16.9)		71.6 (4.2)	**.001**
WASI blocks	22.3 (15.0)		48.3 (12.5)	**.0001**
WASI similarities	23.7 (10.9)		40.95 (6.3)	**.0001**
WASI matrices	14.11 (6.7)		27.5 (9.7)	**.0001**
NART (/50)	28 (3.1)	12/28	48 (1.6)	.0001

**Episodic memory**				
RMT words (/50)	36.25 (7.9)	11/16	47.5 (3.6)	**.0001**
RMT faces (/50)	35.47 (8.4)	11/16	45.2 (4.3)	**.0001**

**Executive function**				
D-KEFS Stroop word	28 (17.6)	6/18	21.5 (4.3)	.65
D-KEFS Stroop inhibition	90.8 (34.9)	16/18	55.5 (10.1)	**.0001**
IGT (net total)	−11.1 (20.5)			

**Semantic processing**				
BPVS (/150)	124.1 (20.1)	19/20	147.8 (1.9)	**.0001**

**Social cognition**				
TASIT (/14)	7.9 (2.5)	16/18	11.75 (1.2)^e^	**.0001**

**Other skills**				
GNT (/30)	11.6 (8.6)	18/18	27 (2.7)	**.0001**
Forwards DS (/12)	8 (2.7)	6/18	8 (2.7)	ns
Reverse DS (/12)	6.5 (2.6)	8/18	6.5 (2.5)	ns
Addition	5.7 (3.5)	7/18	7.1 (2.5)	ns
Subtraction	5.7 (3.4)	8/18	8.2 (2.4)	**.01**
VOSP	15 (4.4)	5/18	18 (1.6)	**.002**

Mean (standard deviation) values shown. Significant group differences in *t* tests *p* < .05 relative to the present control group are shown in bold. BPVS, CBI (item 78 refers to ‘Appears indifferent to the worries and concerns of family members’); D-KEFS Stroop (word and inhibition), Delis-Kaplan Executive Function System; DS, digit span; GNT, Graded Naming Test; IGT, Iowa Gambling Task; n/a, not available; ns, not significant; RMT, recognition memory test; VOSP, Visual Object and Space Perception; WASI, Wechsler Abbreviated Scale of Intelligence.

a CBI information was collected for 12 subjects.

b Defined as <2 years musical training; one control subject had a grade 8 qualification in piano; one patient had a grade 5 qualification in piano.

c One bvFTD patient completed only the mentalising condition of the experimental test and the BPVS; two other patients completed only the experimental test and the BPVS; 14 patients completed the IGT.

d For mean score relative to control group.

e Twelve control subjects completed the TASIT.

**Table 2:** Summary of VBM findings in the bvFTD group.

Behavioural correlate	Direction of correlation	Brain region	Cerebral hemisphere	Peak coordinates	*Z*-score
Mentalising	+	Anterior entorhinal	Right	26 −3 −35	4.09*
Non-mentalising	−	Ventro-medial PFC	Left	−12 69 −9	6.1**
Mentalising > non-mentalising	+	Ventro-medial PFC	Left	−12 69 −9	5.4**

Coordinates (mm) of local maxima for grey matter volume change correlating with behavioural performance in the patient group are shown in Montreal Neurological Institute standard stereotactic space. All results listed were significant at threshold *p* < .05 corrected for multiple comparisons *within the prespecified anatomical small volume of interest (see text), or **over the whole brain.

## Footnotes

Appendix A Supplementary material related to this article can be found at http://dx.doi.org/10.1016/j.cortex.2012.09.011.
