# Supplementary material for: Mentalising music in frontotemporal dementia
Source: Cortex. 2013 Jul;49(7):1844–55. doi: 10.1016/j.cortex.2012.09.011 (PMC3701324; doi:10.1016/j.cortex.2012.09.011)
Supplement: Supplementary file 1 [file mmc1.docx]

**SUPPLEMENTARY MATERIAL**

**Table S1. Music stimuli and foils presented in the experimental behavioural test**

**Mentalising**

| **Trial** | **Musical target (Composer)** | **duration (sec)** | **target** | **close foil** | **distant foil** | **tempo** | **type** |
| --- | --- | --- | --- | --- | --- | --- | --- |
| 1 | Arcana (Varèse) | 30 | adventurous | scolding | dreamy | Moderato | Orchestral |
| 2 | BWV 847 (Bach) | 30 | stressed | secretive | mysterious | Adagio | Piano |
| 3 | Etudes op.10 n°4 (Chopin) | 30 | stressed | secretive | mysterious | Allegro | Piano |
| 4 | Sonate 2 Scherzo (Chopin) | 29 | scolding | adventurous | melancholy | Allegro | Piano |
| 5 | Piano quintet: molto moderato (Fauré) | 31 | mysterious | begging | playful | Allegro | Chamber |
| 6 | Tallis Fantasia (Vaughan Williams) | 32 | mysterious | begging | stressed | Adagio | Orchestral |
| 7 | Phantasiestücke: Langsam (Schumann) | 30 | comforting | upset | heroic | Adagio | Chamber |
| 8 | Lieder Ohne Worte: n°1 (Mendelssohn) | 33 | comforting | upset | heroic | Moderato | Piano |
| 9 | Symphony n°3: 2nd mov (Gorecki) | 30 | dreamy | dreading | adventurous | Adagio | Orchestral |
| 10 | Piano Concerto 1: Larghetto (Chopin) | 31 | dreamy | dreading | adventurous | Adagio | Orchestral |
| 11 | Mantra (Stockhausen) | 30 | dreading | dreamy | comforting | Moderato | Chamber |
| 12 | Music for Strings, Percussion & Celesta: Adagio (Bartok) | 31 | dreading | dreamy | comforting | Adagio | Chamber |
| 13 | Magnificat 1 (Bach) | 29 | heroic | friendly | lazy | Allegro | Orchestral |
| 14 | Gymnopédie 1 (Satie) | 27 | lazy | melancholy | heroic | Adagio | Piano |
| 15 | Appassionata sonata: Andante (Beethoven) | 32 | lazy | melancholy | heroic | Adagio | Piano |
| 16 | Sospiri (Elgar) | 31 | melancholy | lazy | scolding | Adagio | Orchestral |
| 17 | Plink Plank Plunk (Leroy Anderson) | 28 | playful | seductive | begging | Allegro | Orchestral |
| 18 | Tic Toc Choc (Couperin) | 30 | playful | seductive | begging | Allegro | Harpsichord |
| 19 | Piano Concerto 1: Larghetto (Chopin) | 30 | secretive | stressed | seductive | Adagio | Orchestral |
| 20 | Violin Sonata: Blues (Ravel) | 32 | seductive | playful | secretive | Adagio | Chamber |
| Mean (std) |  | 30  (1.4) |  |  |  |  |  |

**Non-mentalising**

| **Trial** | **Musical target (Composer)** | **duration (sec)** | **target** | **close foil** | **distant foil** | **tempo** | **type**** |
| --- | --- | --- | --- | --- | --- | --- | --- |
| 1 | Petite suite en bateau (Debussy) | 26 | bird calls | raindrops | waves | Adagio | Piano |
| 2 | Symphony no 6 Mov 2 (Beethoven) | 18 | bird calls | raindrops | waves | Allegro | Orchestral |
| 3 | Symphony No. 6 opening (Haydn) | 33 | sunrise | waves | storm | Adagio | Orchestral |
| 4 | Ma Mere L’Oye: Le jardin Féerique (Ravel) | 27 | sunrise | waves | storm | Adagio | Orchestral |
| 5 | Revolutionary Etude (Chopin) | 32 | waterfall | flying | horse | Allegro | Piano |
| 6 | Daphnis et Chloé: Suite n°3 (Ravel) | 29 | flying | waterfall | donkey | Adagio | Orchestral |
| 7 | Romeo and Juliet: Death of Tybalt (Prokofiev) | 31 | machinery | car horns | snow | Moderato | Orchestral |
| 8 | Koyaanisqatsi The Grid (Glass) | 34 | machinery | car horns | snow | Allegro | Chamber |
| 9 | Koyaanisqatsi Cloudscape (Glass) | 30 | carhorns | machinery | snow | Adagio | Chamber |
| 10 | Symphony n°4 Scherzo (Tchaïkovsky) | 31 | raindrops | train | bird calls | Allegro | Orchestral |
| 11 | String quartet n°4, mov 4 (Bartok) | 30 | raindrops | train | bird calls | Allegro | Chamber |
| 12 | The Keel Row (Trad) | 29 | horse | donkey | waterfall | Moderato | Piano |
| 13 | German Dance No 3 K605 (Mozart) | 32 | horse | donkey | waterfall | Moderato | Chamber |
| 14 | D’un Jardin Clair (Boulanger) | 31 | snow | waves | machinery | Adagio | Piano |
| 15 | Doctor Gradus ad Parnassum (Debussy) | 24 | snow | waves | machinery | Allegro | Piano |
| 16 | Rite du Printemps: Danse de la terre (Stravinsky) | 23 | storm | train | sunrise | Adagio | Orchestral |
| 17 | The Chairman Dances: Foxtrot (Adams) | 30 | train | storm | sunrise | Moderato | Orchestral |
| 18 | Pacific 231 (Honneger) | 30 | train | storm | sunrise | Adagio | Orchestral |
| 19 | Caravan (Adler) | 15 | wind | waves | car horns | Moderato | Harmonica |
| 20 | Tolstoy farm from Satyagraha (Glass) | 28 | wind | waves | car horns | Allegro | Chamber |
| Mean (std)* |  | 28  (4.9) |  |  |  |  |  |

Trials in each subset were presented in randomised order *mean stimulus duration, tempo and type did not differ significantly (P > 0.05) between subtests

**’type’ here summarises the harmonic and timbral texture of the excerpt (solo instrument, chamber or orchestral)

**Pilot study: selection of music stimuli**

Twenty-five healthy control subjects (18 female) aged 19 – 45 years participated in the pilot study; none subsequently participated in the main study. Most subjects were non-musicians (<2 years musical training on any instrument); five subjects had between three and eight years formal training on an instrument. As a subgroup analysis revealed no significant differences in stimulus matching performance between the non-musician and musically trained subgroups, these were merged for the purpose of selecting music stimuli for the final test. Subjects were presented with 56 Western classical music samples (28 in the mentalising condition, 28 in the non-mentalising condition) in a three alternative, forced-choice word-picture matching paradigm following the same procedure described for the subsequent experimental test (see Methods). From this larger stimulus pool, 20 music samples for which agreement on the represented mental state, object or event was highest among the pilot control group were selected for each condition in the final test. All stimuli included in the final test achieved at least 80% agreement among healthy control subjects. Furthermore, for each of these stimuli, mismatch errors were more frequent with the nominated ‘close’ foil than the nominated ‘distant’ foil for that trial.

**Abbreviations List**

ANOVA Analysis of variance

AUC Area under the curve

bvFTD Behavioural variant frontotemporal dementia

BPVS British Picture Vocabulary Scale

CBI Cambridge Behavioural Inventory

D-KEFS Delis-Kaplan Executive Function System

DS Digit span

FTLD Frontotemporal lobar degeneration

GNT Graded Naming Test

IGT Iowa Gambling Task

MRI Magnetic resonance image

NART National Adult Reading Test

OFC Orbitofrontal cortex

PFC Prefrontal cortex

ROC Receiver operating curve

TASIT The Awareness of Social Inference Test

ToM Theory of mind

RMT Recognition Memory Test

ROC Receiver operating characteristic

VBM Voxel based morphometry

VOSP Visual Object and Space Perception

WASI Weschler Abbreviated Scale of Intelligence
